# Tumor micronecrosis predicts poor prognosis of patients with hepatocellular carcinoma after liver transplantation

**DOI:** 10.1186/s12885-023-10550-w

**Published:** 2023-01-25

**Authors:** Yangyang Wang, Wei Zhang, Hongbin Ge, Xu Han, Jiangchao Wu, Xuqi Sun, Ke Sun, Wanyue Cao, Chao Huang, Jingsong Li, Qi Zhang, Tingbo Liang

**Affiliations:** 1 Department of Hepatobiliary and Pancreatic Surgery, the First Affiliated Hospital, Zhejiang University School of Medicine, Hangzhou, China; 2 Zhejiang Provincial Key Laboratory of Pancreatic Disease, the First Affiliated Hospital, Zhejiang University School of Medicine, Hangzhou, China; 3 Department of Pathology, the First Affiliated Hospital, Zhejiang University School of Medicine, Hangzhou, China; 4 Cancer Center, Zhejiang University, Hangzhou, China; 5 Research Center for Healthcare Data Science, Zhejiang Lab, Hangzhou, China; 6 Zhejiang Clinical Research Center of Hepatobiliary and Pancreatic Diseases, Hangzhou, China; 7 The Innovation Center for the Study of Pancreatic Diseases of Zhejiang Province, Hangzhou, China

**Keywords:** Tumor necrosis, Hepatocellular carcinoma, Liver transplantation, Survival prediction

## Abstract

**Background:**

Tumor micronecrosis is a histopathological feature predicting poor prognosis in patients with hepatocellular carcinoma (HCC) who underwent liver resection. However, the role of tumor micronecrosis in liver transplantation remains unclear.

**Methods:**

We retrospectively reviewed patients with HCC who underwent liver transplantation between January 2015 and December 2021 at our center. We then classified them into micronecrosis(−) and micronecrosis(+) groups and compared their recurrence-free survival (RFS) and overall survival (OS). We identified independent prognostic factors using Cox regression analysis and calculated the area under the receiver operating characteristic curve (AUC) to evaluate the predictive value of RFS for patients with HCC after liver transplantation.

**Results:**

A total of 370 cases with evaluable histological sections were included. Patients of the micronecrosis(+) group had a significantly shorter RFS than those of the micronecrosis(−) group (*P* = 0.037). Shorter RFS and OS were observed in micronecrosis(+) patients without bridging treatments before liver transplantation (*P* = 0.002 and *P* = 0.007), while no differences were detected in those with preoperative antitumor therapies that could cause iatrogenic tumor necrosis. Tumor micronecrosis improved the AUC of Milan criteria (0.77–0.79), the model for end-stage liver disease score (0.70–0.76), and serum alpha-fetoprotein (0.63–0.71) for the prediction of prognosis after liver transplantation.

**Conclusion:**

Patients with HCC with tumor micronecrosis suffer from a worse prognosis than those without this feature. Tumor micronecrosis can help predict RFS after liver transplantation. Therefore, patients with HCC with tumor micronecrosis should be treated with adjuvant therapy and closely followed after liver transplantation.

**Clinical trials registration:**

Not Applicable.

## Introduction

Hepatocellular carcinoma (HCC) is the sixth most common form of cancer worldwide and one of the leading causes of cancer-related mortality with high tumor malignancy [1]. Liver transplantation is a radical treatment for liver cancer, especially early-stage HCC [2]. The shortage of grafts and the risk of recurrence are the two main limitations of liver transplantation in clinical settings; only a few carefully selected patients with HCC can benefit from this treatment [3]. Milan criteria are classical selective standards for defining the eligibility criteria for liver transplantation in patients with HCC having low risks of death and recurrence within 5 years [4]. However, it is too stringent for certain patients with HCC beyond the criteria but would benefit greatly from liver transplantation [5]. Various new selection criteria have been investigated for decades to extend the Milan criteria and optimize the candidate criteria to identify patients who can benefit greatly from liver transplantation, such as the University of California, San Francisco criteria [6], the model for end-stage liver disease (MELD) score [7], and Hangzhou criteria [8]. These criteria are equivalent to or better than the Milan criteria, which can achieve a 4-year OS of 75% [9].

The selected criteria mentioned above mainly include standard tumor parameters of tumor number, size, and biology surrogates such as alpha-fetoprotein (AFP). However, these standards rarely consider the intratumoral features of HCC. We aimed to develop biological behavior markers of intratumoral heterogeneity to better understand this heterogeneous tumor and improve the survival outcomes of patients with HCC. Microscopically observed tumor necrosis is a histopathological feature of the tumor, which is relevant to chronic ischemic injury and angiogenesis [10] [11]. Studies on the effects of necrosis on colorectal cancers [12], cell renal cell carcinomas [13], and breast cancers [14] presented that necrosis is a poor prognostic indicator. Recently, tumor necrosis in HCC was found as a poor prognostic factor for patients with HCC who underwent liver resection [15]. Unlike other published references, we focused on tumor necrosis that can only be observed under the microscopic, and identified this less studied biomarker as ‘tumor micronecrosis’. We found that tumor micronecrosis was also associated with low overall survival rate [16]. Our research previously proposed tumor micronecrosis is a factor in determining adjuvant transcatheter arterial chemoembolization in patients with HCC [17]. Currently, no study on the role of tumor micronecrosis in liver transplantation has been conducted.

This retrospective study aimed to investigate whether tumor micronecrosis and its degree affect the prognosis of patients with HCC who underwent liver transplantation. The results of this study can suggest ideal candidates for liver transplantation and provide predictive values for survival outcomes after liver transplantation in patients with HCC.

## Subjects and methods

### Patients and sample collection

We retrospectively investigated 440 patients diagnosed preoperatively with HCC and who underwent liver transplantation between January 2015 and December 2021 in the First Affiliated Hospital, Zhejiang University School of Medicine (FAHZU). The inclusion and exclusion criteria were as follows: 1) primary histological diagnosis of HCC; 2) underwent liver transplantation; 3) available pathological specimens after surgery. Twenty-one patients with pathologically diagnosed intrahepatic cholangiocarcinoma and 49 patients without pathological specimens were excluded. Consequently, we enrolled 370 patients in our cohort and retrospectively reviewed their electronic medical records for further study. We classified these patients into two groups based on the presence of tumor micronecrosis. The protocol of this study was approved by the ethical committee of FAHZU. All organs were donated voluntarily with written informed consent, and informed consent was obtained from all patients prior to study commencement.

### Histopathologic evaluation of tumor micronecrosis

**Fig. 1:**
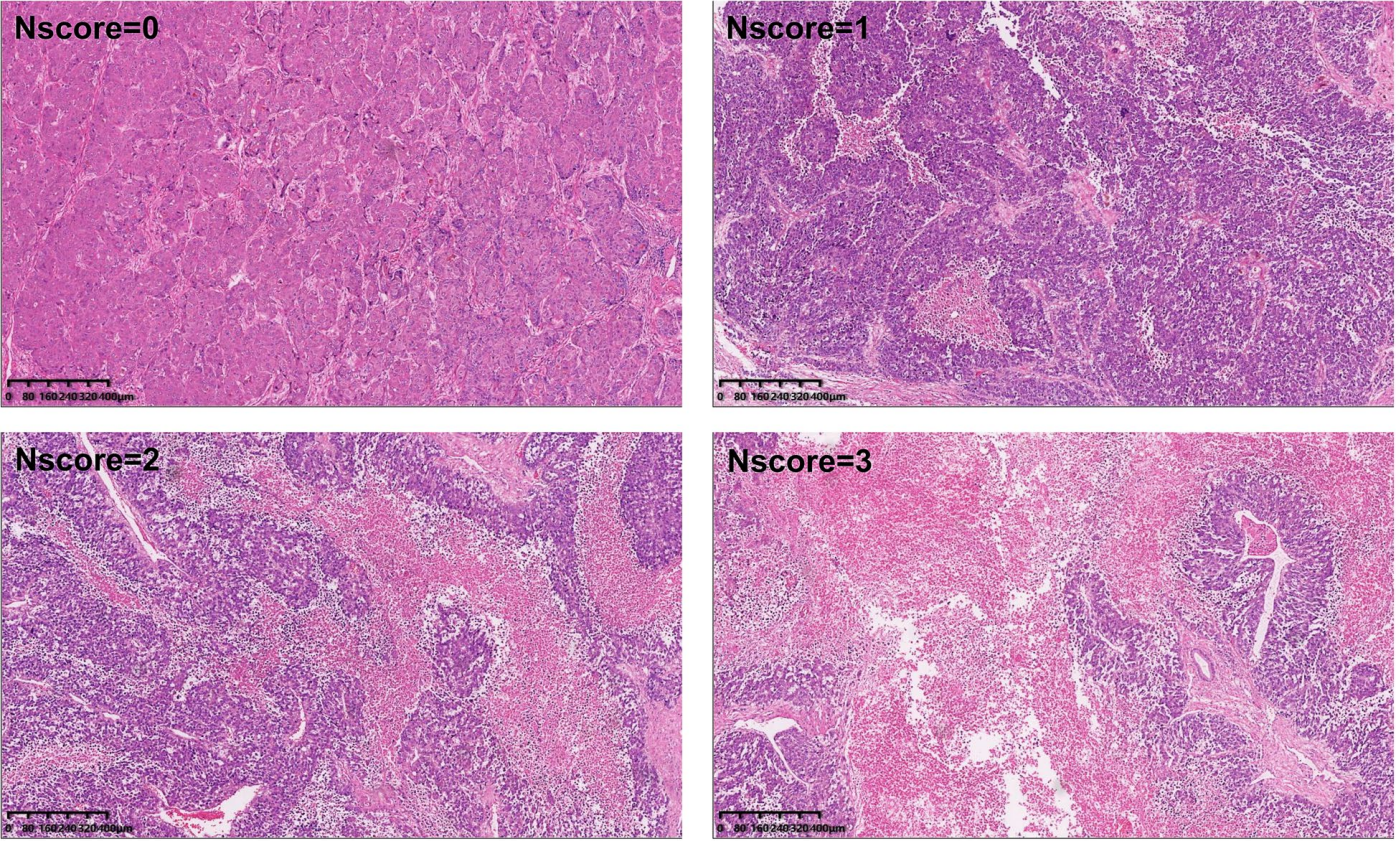
Pathological features of HCC with different Nscores on hematoxylin-eosin staining slides

The pathologic specimen collection was based on the Chinese guidelines for the pathological diagnosis of primary liver cancer: 2015 [18], and used the 7-point baseline sampling protocol. All the pathological tissues were obtained from the explanted livers. When we encountered tissues with gross necrosis led by available treatments before liver transplantation, we selected the section with less bleeding and necrosis and complete tissue, and at least one sample was taken from the central part of the tumor without bleeding and necrosis. For patients with multiple tumors, we chose the 1–3 largest tumors and collected specimens following this protocol as well. All the specimens were embedded in paraffin, cut into slides, and stained using hematoxylin and eosin. A slide imaging system KF-PRO-400 (Konfoong Biotech International Co., LTD. Ningbo, China) was used to scan the sections at a resolution of 0.5 μm/pixel (20X) with a 10X eyepiece magnification, and the images were evaluated at a further 4X magnification using the corresponding software. The experienced pathologists in our center analyzed at least five (one intratumoral zone and 4 periphery of tumor tissues) slides of each specimen, and evaluated the slides for the presence of necrosis and graded the degree of tumor micronecrosis according to the micronecrosis scoring system mentioned previously [17]. The scores reflect the degree of micronecrosis in the tumor: Nscore = 0, negative for necrosis or rarely have micronecrosis (≤ 5%); Nscore = 1, slight micronecrosis (> 5% and ≤ 20%); Nscore = 2, moderate micronecrosis (> 20% and ≤ 50%); Nscore = 3, severe micronecrosis (> 50%) (Fig. 1).

### Follow-up

All patients were followed up after liver transplantation until December 25, 2021, by nurses and physicians in our department. At each follow-up visit, we required the determination of serum tumor markers level every 3 months and radiography examination of computed tomography or magnetic resonance imaging (MRI) every 6 months to detect the recurrence of the tumor. The diagnosis of recurrence of HCC was made by two senior attending surgeons independently and was judged by a third surgeon in case of disagreement. The time from liver transplantation to the first confirmation of tumor recurrence or patient death was defined as recurrence-free survival (RFS). The time from liver transplantation to patient death or the last follow-up was recorded as overall survival (OS).

### Statistical analysis

Statistical analysis was performed using the SPSS v23.0 (IBM, Armonk, NY), R version 4.1.2 for windows, and Prism 8.0 (GraphPad Soft Inc., San Diego, CA) software programs. Categorical variables are summarized using frequencies and percentages and compared using the Chi-squared test or Fisher’s exact test between the micronecrosis(−) and micronecrosis(+) groups. Continuous variables consistent with normal distribution are expressed as the mean ± standard deviation. As appropriate, the Student’s *t*-test or the Mann-Whitney *U* test was used for comparison. Variables inconsistent with normal distribution are presented as median (range) values and compared using the Mann-Whitney *U* test. The association between tumor micronecrosis and baseline characteristics as well as correlation between tumor micronecrosis and preoperative treatment was evaluated by Pearson correlation analysis. Cumulative OS and RFS rates were calculated using the Kaplan–Meier method and compared using the log-rank test; *P* <  0.05 was considered statistically significant. Univariate and multivariate analyses were performed to identify independent factors affecting RFS. Logistic regression and k-nearest neighbor (kNN) algorithms were implemented to construct the prediction models [19, 20]. The utility of prediction models for RFS after liver transplantation was evaluated by using the receiver operating characteristic (ROC) curve, and an area under the ROC curve (AUC) of more than 0.7 was considered to be clinically useful [21].

## Results

### Patient characteristics

**Fig. 2:**
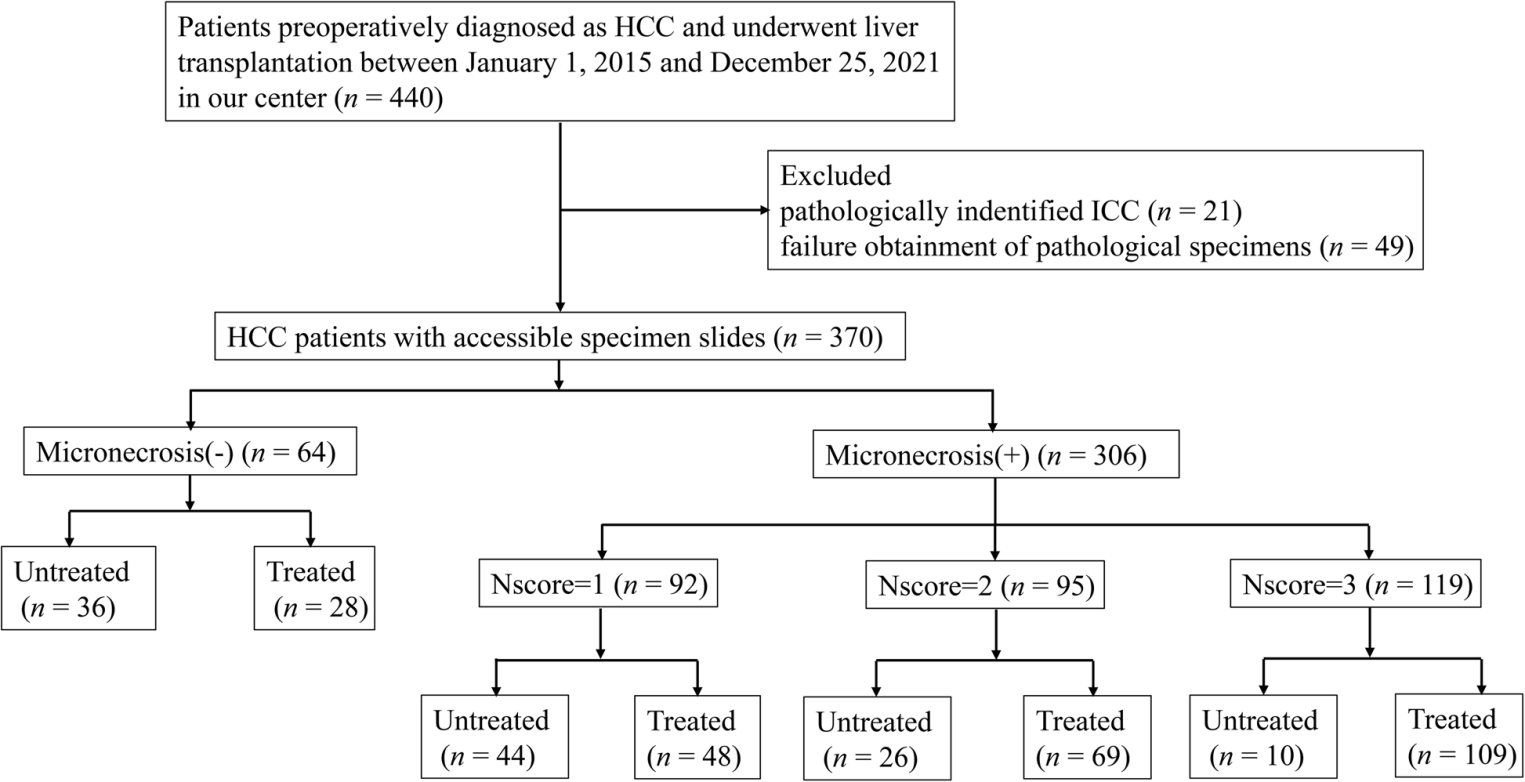
Enrollment flowchart of the cohort

A total of 370 patients with HCC who underwent liver transplantation at our center were enrolled in the analytic cohort, and the enrollment flowchart is presented in Fig. 2. The mean age of all patients was 52.9 ± 9.0 years at the time of liver transplantation, and the majority of patients were male (*n* = 328, 88.6%). Most patients had hepatitis B (*n =* 337, 91.1%) and cirrhosis (*n* = 357, 96.5%). About three-quarters of the patients had no more than three tumors (*n* = 286, 77.3%), and the largest tumor was usually no more than 5 cm (*n* = 267, 72.2%). Most patients had compensatory liver function manifested as Child-Pugh class A and B (*n* = 310, 83.8%). Nearly half of the patients exceeded the Milan criteria (*n* = 194, 52.4%).

Our study identified 64 patients in the micronecrosis(−) group and 306 patients in the micronecrosis(+) group (Nscore = 1, *n* = 92; Nscore = 2, *n* = 95; Nscore = 3, *n* = 119). The demographic and clinicopathological characteristics of the two groups are shown (Table 1). We followed up the patients with a median of 32.2 months (range: 0.03–85.0 months). After excluding patients who had received preoperative treatment, 80 of 116 HCC patients were present with tumor micronecrosis (Table 2).

**Table 1:** Demographics and clinicopathological characteristics of patients with HCC receiving liver transplantation

Characteristics	Total (*n* = 370)	Micronecrosis (−) (*n* = 64)	Micronecrosis (+) (*n* = 306)	*P*-value
Age, years	52.9 ± 9.0	52.9 ± 9.1	52.9 ± 9.0	0.993
Gender, *n* (%)				0.106
Male	328 (88.6%)	53 (83.8%)	275 (89.9%)	
Female	42 (11.4%)	11 (16.2%)	31 (10.1%)	
Etiology, *n* (%)				0.011
HBV infection	337 (91.1%)	53 (82.8%)	284 (92.8%)	
others	33 (8.9%)	11 (17.2%)	22 (7.2%)	
Cirrhosis, *n* (%)	357 (96.5%)	63 (98.4%)	294 (96.1%)	0.707*
AFP, ng/mL				0.354
AFP ≤400	278 (75.1%)	51 (79.7%)	227 (74.2%)	
AFP > 400	92 (24.9%)	13 (20.3%)	79 (25.8%)	
RDW	15.3 ± 2.8	16.0 ± 3.0	15.2 ± 2.7	0.035
NLR	4.80 ± 5.48	3.81 ± 4.32	4.89 ± 5.68	0.150
INR	1.28 ± 0.28	1.39 ± 0.30	1.25 ± 0.28	0.001
TNM stage, *n* (%)				< 0.001*
I	137 (37.0%)	39 (60.9%)	98 (32.0%)	
II	128 (34.6%)	17 (26.6%)	111 (36.3%)	
III	90 (24.3%)	8 (12.5%)	82 (26.8%)	
IV	15 (4.1%)	0 (0.0%)	15 (4.1%)	
Tumor number, *n* (%)				0.070
≤3	286 (77.3%)	55 (85.9%)	231 (75.5%)	
> 3	84 (22.7%)	9 (14.1%)	75 (24.5%)	
Size of largest tumor, cm				0.003
≤5	267 (72.2%)	56 (87.5%)	211 (69.0%)	
> 5	103 (27.8%)	8 (12.5%)	95 (31.0%)	
Differentiation, *n* (%)				0.023*
Well	38 (10.3%)	7 (10.9%)	31 (10.1%)	
Moderate	197 (53.2%)	41 (64.1%)	156 (51.0%)	
Poor	128 (34.6%)	13 (20.3%)	115 (37.6%)	
NA	7 (1.9%)	3 (4.7%)	4 (1.3%)	
Macrovascular invasion, *n* (%)				0.015
Present	90 (24.3%)	8 (12.5%)	82 (26.8%)	
Absent	280 (75.7%)	56 (87.5%)	224 (73.2%)	
Child-Pugh class, *n* (%)				0.047
A (5–6)	159 (43.0%)	19 (29.7%)	140 (45.8%)	
B (7–9)	151 (40.8%)	34 (53.1%)	117 (38.2%)	
C (≥ 10)	60 (16.2%)	11 (17.2%)	49 (16.0%)	
Milan criteria, *n* (%)				< 0.001
Within	176 (47.6%)	45 (70.3%)	131 (42.8%)	
Out	194 (52.4%)	19 (29.7%)	175 (57.2%)	
MELD score	11 (6–40)	14 (6–28)	10 (6–40)	0.011
Preoperative treatment, *n* (%)				< 0.001
Yes	254 (68.6%)	28 (43.8%)	226 (73.9%)	
Liver resection	18 (4.9%)	4 (6.3%)	14 (4.6%)	0.529*
TACE	80 (21.6%)	9 (14.1%)	71 (23.2%)	0.106
RFA	15 (4.1%)	0	15 (4.9%)	0.048*
Targeted therapy	2 (0.5%)	0	2 (0.7%)	1.000*
Radiotherapy	3 (0.8%)	1 (1.6%)	2 (0.7%)	0.528*
Other	19 (5.1%)	2 (3.1%)	17 (5.6%)	0.548*
multiple	117 (31.6%)	12 (18.8%)	105 (34.3%)	0.015
No	116 (31.4%)	36 (56.3%)	80 (26.1%)	
Recurrence, *n* (%)	109 (29.5%)	11 (17.2%)	98 (32.0%)	0.018
Intrahepatic	40 (36.7%)	1 (9.1%)	39 (39.8%)	0.084
Thorax	43 (39.4%)	2 (18.2)	41 (41.8%)	0.115
Bone	19 (17.4%)	3 (27.3)	16 (16.3%)	0.390
Abdominal cavity	40 (36.7%)	6 (54.4%)	34 (34.7%)	0.184
Multiple	29 (26.6%)	1 (9.1%)	28 (28.6%)	0.274
Others	7 (6.4%)	1 (9.1%)	6 (6.1%)	
Death, *n* (%)	116 (31.4%)	17 (26.6%)	99 (32.4%)	0.364

^*^ Fisher exact test was performed for the comparison

AFP, alpha-fetoprotein; RDW, red cell volume distribution width; NLR, neutrophil-to-lymphocyte ratio; INR, international normalized ratio; MELD, model for end-stage liver disease

**Table 2:** Baseline characteristics of HCC patients receiving liver transplantation without preoperative treatment

Characteristics	Total (*n* = 116)	Micronecrosis (−) (*n* = 36)	Micronecrosis (+) (*n* = 80)	*P*-value
Age, years	52.0 ± 9.3	52.6 ± 9.7	51.7 ± 9.1	0.618
Gender, *n* (%)				0.400
Male	104 (88.6%)	31 (86.1%)	73 (91.3%)	
Female	12 (10.3%)	5 (13.9%)	7 (8.8%)	
Etiology, *n* (%)				0.077
HBV infection	100 (86.2%)	28 (77.8%)	72 (90.0%)	
others	16 (13.8%)	8 (22.2%)	8 (10.0%)	
Cirrhosis, *n* (%)	113 (97.4%)	35 (97.2%)	78 (97.5%)	1.000*
AFP, ng/mL, *n* (%)				0.178
AFP ≤400	91 (78.4%)	31 (86.1%)	60 (75.0%)	
AFP > 400	25 (21.6%)	5 (13.9%)	20 (25.0%)	
RDW	15.8 ± 3.1	16.2 ± 3.1	15.6 ± 3.1	0.312
NLR	4.39 ± 5.53	4.60 ± 6.03	4.89 ± 5.68	0.529
INR	1.41 ± 0.33	1.46 ± 0.29	1.39 ± 0.35	0.305
TNM stage, *n* (%)				< 0.001*
I	81 (69.8%)	32 (88.9%)	49 (61.3%)	
II	2 (1.7%)	2 (5.6%)	0 (0.0%)	
III	33 (28.4%)	2 (5.6%)	31 (38.8%)	
Tumor number, *n* (%)				0.011
≤3	93 (80.2%)	34 (94.4%)	59 (73.8%)	
> 3	23 (19.8%)	2 (5.6%)	21 (26.3%)	
Size of largest tumor, cm				0.059
≤5	83 (71.6%)	30 (87.5%)	53 (66.3%)	
> 5	33 (28.4%)	6 (12.5%)	27 (33.8%)	
Differentiation, *n* (%)				0.020*
Well	5 (4.3%)	4 (11.1%)	1 (1.3%)	
Moderate	71 (61.2%)	26 (72.2%)	45 (56.3%)	
Poor	36 (31.0%)	5 (13.9%)	31 (38.8%)	
NA	4 (3.4%)	1 (2.8%)	3 (3.8%)	
Macrovascular invasion, *n* (%)				0.003
Present	31 (26.7%)	3 (8.3%)	28 (35.0%)	
Absent	85 (73.3%)	33 (91.7%)	52 (65.0%)	
Child-Pugh class, *n* (%)				0.987
A (5–6)	30 (25.9%)	9 (25.0%)	21 (26.3%)	
B (7–9)	60 (51.7%)	19 (52.8%)	41 (51.2%)	
C (≥ 10)	26 (22.4%)	8 (22.2%)	18 (22.5%)	
Milan criteria, *n* (%)				< 0.001
Within	61 (52.6%)	28 (77.8%)	33 (41.3%)	
Out	55 (47.4%)	8 (22.2%)	47 (58.8%)	
MELD score	11 (6–40)	14 (6–28)	10 (6–40)	0.758
Recurrence, *n* (%)	41 (35.3%)	6 (16.7%)	35 (43.8%)	0.005
Death, *n* (%)	32 (27.6%)	5 (13.9%)	27 (33.8%)	0.027

### Baseline comparison of micronecrosis(+) and micronecrosis(−) groups

Patients with tumor micronecrosis were more likely to have advanced tumor-node-metastasis (TNM) stage (*P* <  0.001), larger tumor size (*P* = 0.003), poorer differentiation (*P* = 0.023), and more frequent macrovascular invasion (*P* = 0.015). More patients in the micronecrosis(+) group exceeded Milan criteria and received preoperative treatments (*P* <  0.001 for both) than patients in the micronecrosis(−) group, and the MELD score was lower in the micronecrosis(+) group than in the micronecrosis(−) group (*P* = 0.011). Patients in the micronecrosis(+) group developed hepatitis B virus infection more often than those in the micronecrosis(−) group (*P* = 0.011), had lower red cell volume distribution width (RDW) and international normalized ratio (INR) (*P* = 0.035 and 0.001, respectively) (Table 1).

In patients without preoperative treatment, patients with micronecrosis had more tumor nodules (*P* = 0.011), poorer differentiation (*P* = 0.020) and more frequent macrovascular invasion (*P* = 0.003) than patients without tumor micronecrosis. More patients in the micronecrosis(+) group exceeded Milan criteria and had a higher TNM stage than the micronecrosis(−) group (both *P* <  0.001) (Table 2). Moreover, the presence of tumor micronecrosis was associated with TNM stage (*r* = 0.312, *P* = 0.001, Pearson’s chi-squared test), tumor nodules (*r* = 0.240, *P* = 0.009), tumor differentiation (*r* = 0.223, *P* = 0.018), macrovascular invasion (*r* = 0.279, *P* = 0.002), and Milan criteria (*r* = 0.338, *P* <  0.001).

### Survival outcomes of micronecrosis(+) and micronecrosis(−) groups

**Fig. 3:**
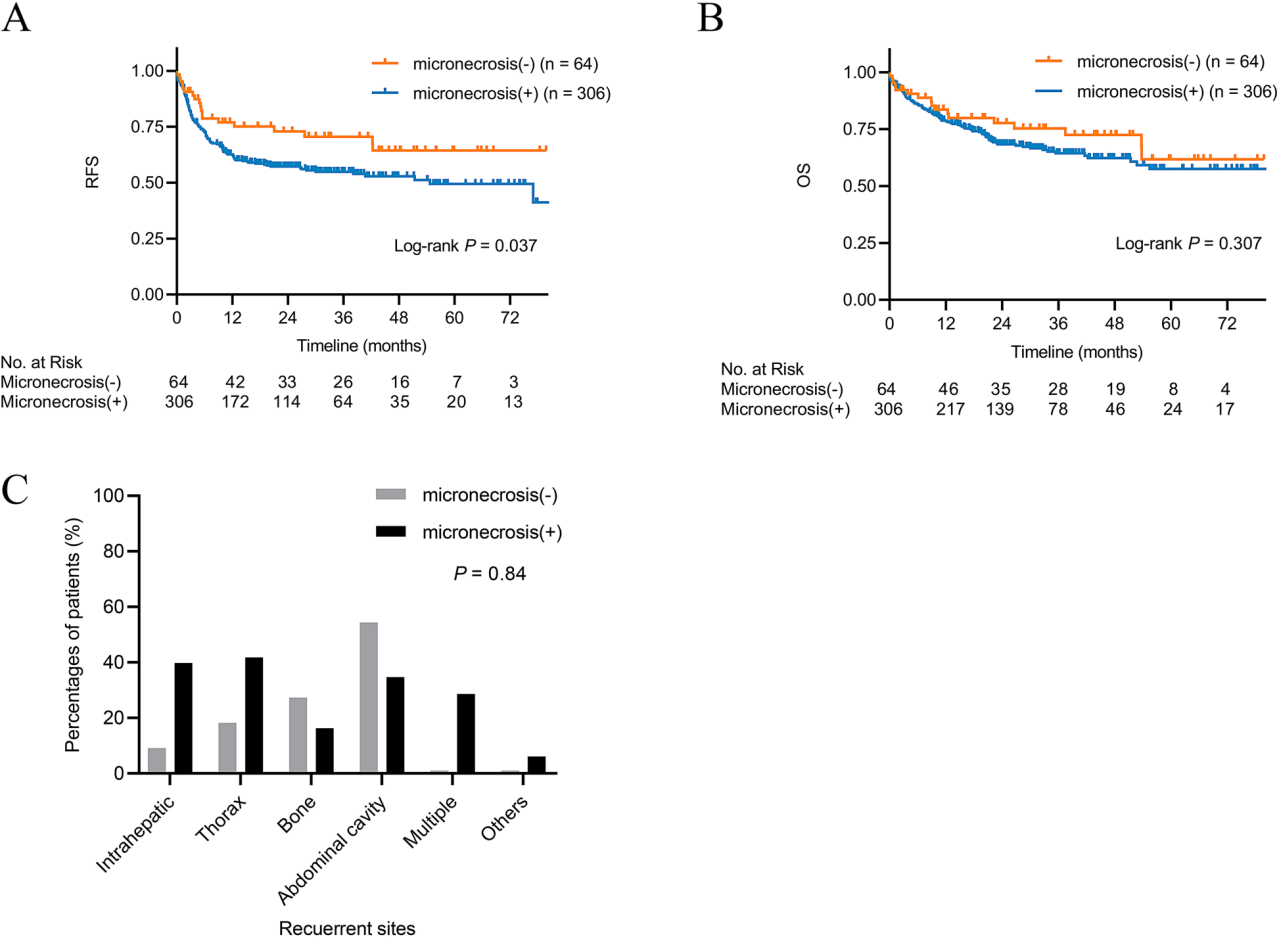
Recurrence-free survival (**A**) and overall survival (**B**) after liver transplantation in patients with hepatocellular carcinoma in the micronecrosis(−) and micronecrosis(+) groups. (**C**) Percentages of recurrence sites in the two groups

The median follow-up was 32.2 months. During the follow-up, recurrence occurred in 11 patients (17.2%) from the micronecrosis(−) group and 98 patients (32.0%) from the micronecrosis(+) group (*P* = 0.018). The 1-, 3-, and 5-year RFS rates in the micronecrosis(−) group were 77.0, 70.5, and 64.4%, respectively, which were higher than those in the micronecrosis(+) group, 62.4, 54.7, and 49.3%, respectively (*P* = 0.037) (Fig. 3A). Seventeen patients (26.6%) in the micronecrosis (−) group and 99 patients (32.4%) in the micronecrosis(+) group died (*P* = 0.364). No significant difference in the 1-, 3-, and 5-year OS rate was observed between patients with tumor micronecrosis (78.7, 64.4, and 57.5%) and those without tumor micronecrosis (83.6, 75.3, and 61.7%, *P* = 0.307) (Fig. 3B). Tumors recurred less frequently in intrahepatic sites in the micronecrosis(−) patients; local recurrence was found in one patient (9.1%) in the micronecrosis(−) group and 39 patients (39.8%) in the micronecrosis(+) group (*P* = 0.84) (Fig. 3C).

**Fig. 4:**
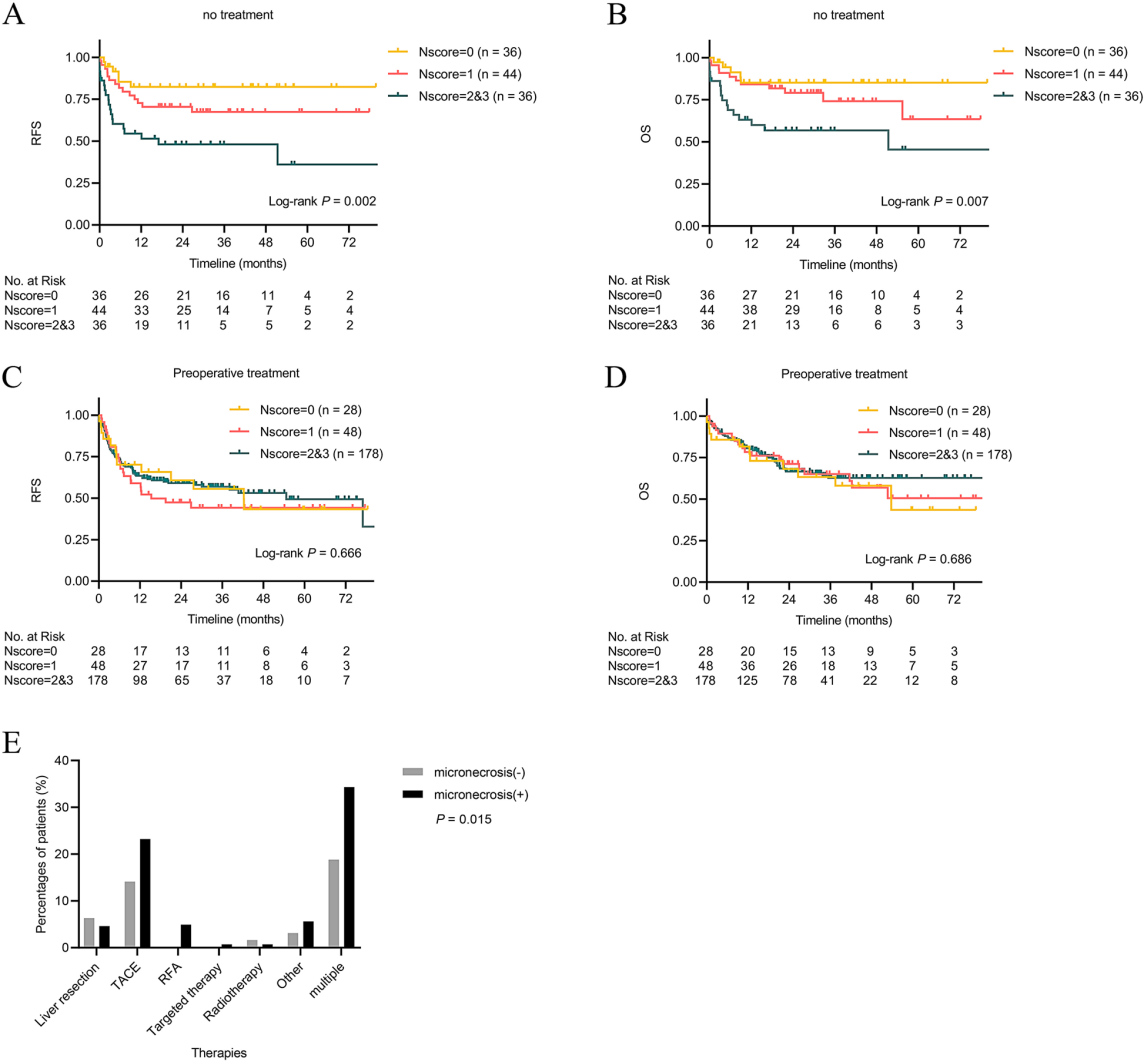
Recurrence-free survival (**A**) and overall survival (**B**) after liver transplantation in patients with hepatocellular carcinoma without preoperative antitumor treatment under different degrees of tumor micronecrosis. Recurrence-free survival (**C**) and overall survival (**D**) after liver transplantation in patients with HCC undergoing preoperative treatment under different degrees of tumor micronecrosis. (E) Details of preoperative treatment in patients

We observed even more significant differences in prognosis between patients with and without tumor micronecrosis among untreated patients in our classified population-based study depending on whether they had preoperative antitumor treatment. The 1-, 3-, and 5-year RFS rates in patients with tumor micronecrosis were 62.1, 56.3, and 50.6%, respectively, which were much lower than 82.3, 82.3, and 82.3%, respectively, in patients without tumor micronecrosis (*P* = 0.016). The 1-, 3-, and 5-year OS rates in the micronecrosis(+) group (76.6, 67.8, and 56.5%) were poorer than those in the micronecrosis(−) group (84.8, 84.8, and 84.8%, *P* = 0.040). The degree of tumor micronecrosis was also associated with RFS (Nscore = 0 vs. Nscore = 1 vs. Nscore = 2 and 3, *P* = 0.002) and OS (Nscore = 0 vs. Nscore = 1 vs. Nscore = 2 and 3, *P* = 0.007) (Fig. 4A and B). The prognosis of high-score necrosis was poorer than the low-score necrosis.

However, there was no difference in the prognosis between patients with and without tumor micronecrosis who received preoperative treatments. The 1-, 3-, and 5-year RFS rates in the micronecrosis(−) group were 70.1, 60.7, and 43.3%, respectively, which were comparable to 63.5, 54.8, and 49.5%, respectively, in the micronecrosis(+) group (*P* = 0.999). The 1-, 3-, and 5-year OS rates in the micronecrosis(−) group (81.6, 63.3, and 43.5%, respectively) were similar to those in the micronecrosis(+) group (80.1, 63.4, and 58.5%, respectively; *P* = 0.416). We also did not find significant difference of RFS or OS based on Nscores (*P* = 0.666 and 0.686, respectively) (Fig. 4C and D).

To explain this phenomenon, we analyzed the preoperative treatment conditions of patients (Table 1 and Fig. 4E). We did not find significant difference in therapies of liver resection, transcatheter arterial chemoembolization (TACE), radiofrequency ablation (RFA), targeted therapy and radiotherapy alone in patients with tumor micronecrosis; however, more patients in the micronecrosis(+) group received comprehensive treatment (*P* = 0.015). We also evaluated the relationship between preoperative treatment and tumor micronecrosis, and correlation analysis showed that preoperative antitumor treatment was positively correlated with tumor micronecrosis (*r* = 0.245, *P* <  0.001, Pearson correlation). The result indicated that multiple bridging treatments may affect necrosis scores, and we should analyze patients differently according to their treatment situation.

### Independent risk factors for the prognosis of patients without preoperative treatment

We carefully chose the variables to establish the Cox regression model of RFS. After univariate analysis, variables with *P* <  0.1 of serum AFP level, neutrophil-to-lymphocyte ratio, tumor number, size of the largest tumor, tumor differentiation, macrovascular invasion, Child-Pugh class, and tumor micronecrosis were included in the multivariate Cox regression model. In terms of RFS, we identified tumor number ≥ 3 (HR, 2.905; 95% CI, 1.227–6.875; *P* = 0.015) and advanced Child-Pugh class (HR, 6.692; 95% CI, 2.012–22.262; *P* = 0.004) as independent prognostic factors in patients with HCC after liver transplantation. Even though tumor micronecrosis increased the possibility of poor prognosis, it was not an independent prognostic factor after liver transplantation (Table 3).

**Table 3:** Univariate and multivariate analyses of prognostic factors of RFS in patients without preoperative anti-tumor treatment

	Univariate analysis		Multivariate analysis	
Variables	HR (95% CI)	*P*-value	HR (95% CI)	*P*-value
Age	1.005 (0.972–1.039)	0.776		
Gender	2.440 (0.589–10.116)	0.219		
HBV infection	1.659 (0.591–4.656)	0.336		
AFP > 400 ng/mL	1.909 (0.986–3.694)	0.055	1.032 (0.451–2.363)	0.941
RDW	1.067 (0.973–1.170)	0.166		
NLR	1.052 (1.019–1.086)	0.002	1.022 (0.983–1.062)	0.273
INR	0.906 (0.340–2.410)	0.843		
Size of the largest tumor	2.885 (1.556–5.347)	0.001	2.115 (0.996–4.495)	0.051
Tumor number	4.809 (2.558–9.040)	< 0.001	2.905 (1.227–6.875)	0.015
Poor differentiation	4.032 (2.121–7.665)	< 0.001	1.894 (0.895–4.009)	0.095
Macrovascular invasion	4.521 (2.429–8.415)	< 0.001	2.360 (0.919–6.058)	0.074
Child-Pugh class		0.039		0.004
A (5–6)	Reference		Reference	
B (7–9)	2.606 (0.990–6.861)	0.052	6.692 (2.012–22.262)	0.002
C (≥ 10)	3.834 (1.364–10.776)	0.011	8.160 (2.279–29.212)	0.001
Micronecrosis	3.021 (1.270–7.184)	0.012	1.482 (0.588–3.735)	0.404

### Tumor micronecrosis adjusted to prognosis models

**Fig. 5:**
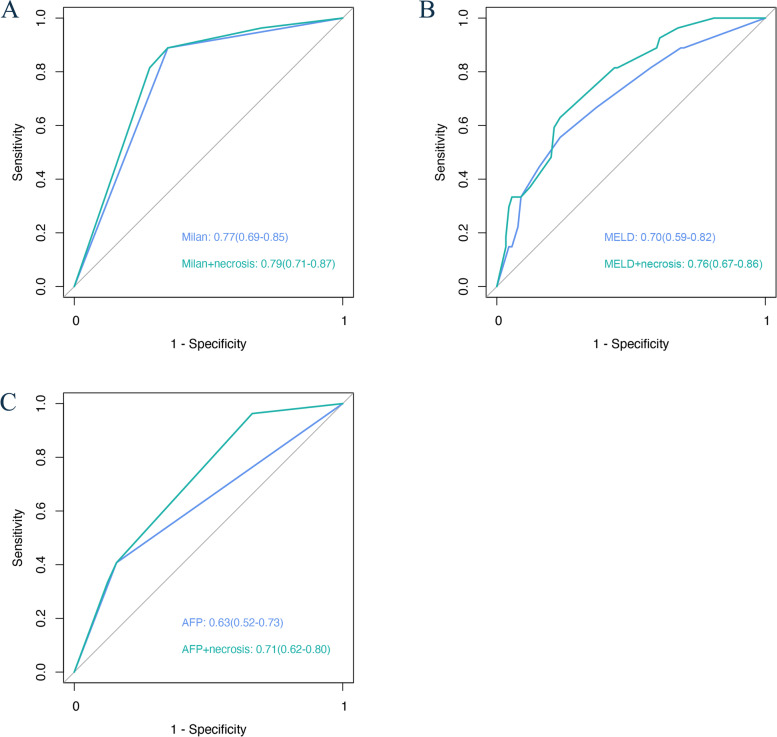
Receiver operating characteristic curve of prediction models of 5-year recurrence-free survival for patients with hepatocellular carcinoma without preoperative antitumor treatment ROC curve performed by k-Nearest Neighbors algorithm of (**A**) Milan criteria vs. Milan criteria incorporated with tumor micronecrosis, (**B**) MELD score vs. MELD score incorporated with tumor micronecrosis, and (**C**) AFP level vs. AFP level incorporated with tumor micronecrosis.

We used tumor micronecrosis to illustrate the potential incremental value above the frequently used survival prediction models of liver transplantation. Models incorporating micronecrosis displayed better predictive accuracy in predicting 5-year RFS in patients with HCC after liver transplantation than models based on the Milan criteria, MELD score, and AFP level alone. We evaluated the performance of models by logistic regression and k-nearest neighbor algorithms. For adjusted Cox regression models, tumor micronecrosis improved the AUC value of Milan criteria from 0.705 to 0.738, the AUC value of MELD score from 0.682 to 0.738, and the AUC value of AFP level from 0.566 to 0.664. The former two models had moderate accuracy and were considered clinically useful. What’s more, kNN exhibited a superior performance showed as AUC values of 0.79, 0.76 and 0.71 (Fig. 5A-C).

## Discussion

Tumor necrosis has been shown to be a marker of poor prognosis of solid tumors [22], and a similar conclusion in HCC was confirmed recently [23]. Nevertheless, no study has reported the effect of necrosis on liver transplantation in patients with HCC yet. In this retrospective study, we found that patients with HCC who underwent transplantation but showed no tumor micronecrosis have a longer RFS than those with tumor micronecrosis. Patients with HCC without tumor micronecrosis could be more suitable candidates for liver transplantation. Baseline differences of more advanced TNM stage, larger tumor size, poorer differentiation, and more frequent macrovascular invasion in the micronecrosis(+) group suggested that tumor micronecrosis was associated with the tumor’s higher malignant biology more likely to recur after liver transplantation. Therefore, we explored its selective and predictive values in liver transplantation recipients. Although tumor micronecrosis was not a decisive, independent prognostic factor in the multivariate analysis, it improved the prediction ability of RFS after liver transplantation in the adjustment models.

The recipients often underwent complex antitumor treatments before liver transplantation, complicating the situation. The bridging treatments, mainly combined with TACE and RFA [24], can lead to tumor necrosis simultaneously [25] while achieving an excellent curative effect in patients on the waiting list and down-staging tumors to meet the standard of liver transplantation [26]. Necrosis caused by antitumor treatment typically presents as large-area necrosis and can be observed radiologically and by gross evaluation [27]. We also found a weak positive correlation between preoperative treatment and tumor micronecrosis. In our further analysis of subgroups based on treatment statuses, we found that micronecrosis(+) patients had an even worse prognosis than micronecrosis(−) patients in the untreated group. At the same time, there was no significant difference in the prognosis of the treated group. The variability in outcome analysis also indicated that tumor micronecrosis is a biological behavior. Biological behavior directly determines the recurrence after transplantation [28], and the newly identified tumor micronecrosis in HCC is a potential one. The necrosis caused by preoperative antitumor therapies was not related to the biological behavior of HCC; however, it can interfere with our judgment of micronecrosis. Therefore, the iatrogenic tumor necrosis cannot reflect the biological behavior of tumors, and the treating modalities can also influence the prognosis [29], leading to a poor prediction effect. Thus, it is worthwhile to explore the internal mechanism of tumor micronecrosis in HCC. When excluding patients with bridging treatment, we found that tumor micronecrosis influenced prognosis greatly in patients with HCC undergoing liver transplantation, but the degree of tumor micronecrosis can help stratify the survival outcomes. These findings stressed that the significant application of tumor micronecrosis is to eliminate the interference of antitumor therapy. Tumor micronecrosis plays a substantial role in the prognosis for patients without preoperative treatment, while the impact on patients receiving bridging treatments should be carefully considered.

Liver transplantation is the optimal therapy for treating HCC and cirrhosis concurrently [30], and cirrhosis was present in most patients in our cohort. The risk of recurrence after liver transplantation cannot be ignored. In recent years, expansion of the Milan criteria has been a research focus to facilitate the allocation of grafts to patients with a low risk of recurrence. Novel predictive factors and models were explored to select the best candidate groups for liver transplantation. For example, pretransplant serum AFP was a biomarker to predict outcomes of patients with HCC after liver transplantation [31]. The microvascular invasion was an independent predictor of HCC recurrence after liver transplantation [32], and the status of microvascular invasion was predicted by tools such as gadoxetic acid-enhanced MRI [33]. MELD score served as a metric for the survival benefit of liver transplantation [34]. A metroticket 2.0 model has been used to analyze the risk of death after liver transplantation for patients with HCC [35]. A combination of these factors could also predict the recurrence risk after liver transplantation [36, 37]. We found improvements in the prediction efficiency of Milan criteria, MELD score, and AFP level when incorporating tumor micronecrosis. These adjusted models showed satisfactory prediction efficacy, and could define patients with a high risk of short RFS after liver transplantation to provide guidance for postoperative adjuvant therapy in patients with an increased risk of recurrence and death.

Two theories may elucidate the mechanistic relationship between tumor micronecrosis and prognosis. One is based on the conclusion that immunosuppression is an oncogenesis factor of HCC [38]. Tumor necrosis is functionally mediated by infiltrating monocytes/macrophages [39, 40], and it has been demonstrated that the mobilization of monocytic myeloid-derived suppressor cells (MDSCs) promotes tumor recurrence after liver transplantation via CXCL10/TLR4/MMP14 signaling [41]. Ouzounova et al. have shown that tumor-infiltrated monocytic MDSCs facilitate tumor cell dissemination from the primary site by inducing epithelial-mesenchymal transition (EMT)/cancer stem cell (CSC) phenotype. At the same time, granulocytic MDSC infiltrates support the metastatic growth by reverting EMT/CSC phenotype and promoting tumor cell proliferation [42]. In our cohort, recurrence was more frequent in the micronecrosis(+) group. Therefore, we inferred that tumor micronecrosis represents MDSC infiltration and may lead to a high recurrence rate after liver transplantation. The characteristics of the immune microenvironment are worth studying. Another theory pointed at the hypoxia environment of micronecrosis. Rapidly dividing tumor cells create a hypoxic environment [43] and force the tumor to adapt to hypoxia by changing its gene expression pattern to resist hypoxia-induced cell death [44]. The macrophages exposed to hypoxia accumulate HIF1α and HIF2α [45], thereby promoting tumor growth through the controlled PI3K signaling pathway [46].

This study has several limitations. First, the complex bridging treatment before liver transplantation was a nonnegligible interference factor to our judgment and score of tumor micronecrosis. Although we can distinguish between iatrogenic massive necrosis and tumor micronecrosis as mentioned above, there is no obvious mechanistic explanation between treatment and tumor micronecrosis, and the mechanism as well as the discrimination of tumor pathological micronecrosis and slight iatrogenic necrosis are still under our investigation. Nevertheless, it does not affect our conclusions for patients who have not received preoperative antitumor treatment. Second, this was a single-center, retrospective study. Thus, we must validate our results through a multicenter, prospective clinical trial; however, the volume of liver transplantation cases in other centers is small, remaining the weakness in establishing an external validation set to verify our adjusted model. Due to the importance of external validation and the difficulty of data acquisition, this work will continue in our future research. Third, it is hard to detect tumor micronecrosis before surgery, limiting the clinical application value of tumor micronecrosis in candidate selection for liver transplantation. We are investigating two strategies to accurately quantify this biomarker before surgery. One uses needle biopsy samples of tumors to evaluate tumor micronecrosis. The other is through the use of models to predict the status of tumor micronecrosis by combining blood test results and radiomics features before surgery.

In conclusion, this study showed that patients with HCC without tumor micronecrosis could achieve a better prognosis after liver transplantation than those with tumor micronecrosis. Tumor micronecrosis can improve the predictive efficiency of Milan criteria, MELD score, and AFP level. These findings can predict the long-term RFS of patients with HCC after liver transplantation, as well as guiding the application of adjuvant therapy for patients with HCC and close follow-up after liver transplantation.

## Data Availability

The datasets used during the current study are available from the corresponding author on reasonable request.

## Abbreviations

HCC: hepatocellular carcinoma
RFS: recurrence-free survival
OS: overall survival
AUC: area under the receiver operating characteristic curve
MELD: model for end-stage liver disease
AFP: alpha-fetoprotein
MRI: magnetic resonance imaging
ROC: the receiver operating characteristic
TNM: tumor-node-metastasis
TACE: therapies of transcatheter arterial chemoembolization
RFA: radiofrequency ablation
MDSCs: myeloid-derived suppressor cells
EMT: epithelial-mesenchymal transition
CSC: cancer stem cell
